# Hepatotoxicity in patients co-infected with HIV and tuberculosis while receiving NNRTI-based antiretroviral regimen and rifampicin

**DOI:** 10.1186/1758-2652-13-S4-P90

**Published:** 2010-11-08

**Authors:** W Mankhatitham, A Lueangniyomkul, W Manosuthi

**Affiliations:** 1 Bamrasnaradura Infectious Diseases Institute, Nonthaburi, Thailand

## Objective

To evaluate the rate of and risk factors associated with hepatotoxicity in tuberculosis (TB) and human immunodeficiency virus type 1 (HIV-1) co-infected patients while receiving nonnucleoside reverse transcriptase inhibitors (NNRTI)-based antiretroviral therapy (ART) and rifampicin (RMP)-containing anti-TB regimens.

## Methods

We analyzed data from an open label, randomized, comparative trial comparing treatment outcome between 71 TB/HIV-1 co-infected patients receiving efavirenz (EFV)-based and nevirapine (NVP)-based ART and all were receiving RMP containing anti-TB regimens. Demographic data, liver function profile, CD4 cell count, plasma HIV-1 RNA, hepatitis B surface antigen and anti- hepatitis C virus antibody were collected before initiating ART (week 0). Liver enzymes and total bilirubin level were monitored at 6 weeks, 12 weeks and 24 weeks after ART initiation. All patients were followed until TB therapy was completed or 24 weeks after ART initiation if TB therapy was not completed.

## Results

Of 134 patients, mean (SD) age was 36.8 ± 8.6 years and 67.2% were male. HCV co-infection was found in 23.9% of patients. Severe hepatotoxicity (grade 3 or 4) developed in 4 (23.9%) patients; 3 patients in NVP group and 1 patient in EFV group (P=0.355). Severe hyperbilirubinemia (grade 3 or 4) occurred in 5 (7.7%) patients in NVP group and 2 (2.9%) patients in EFV group (P=0.264). By univariate analysis, cotrimoxazole use (OR, 2.65; 95%CI, 0.99-7.02) and HCV co-infection (OR, 3.40; 95%CI, 1.49-7.37) were associated with grade 1-4 hepatotoxicity. By multivariate analysis, HCV co-infection (AOR, 3.03; 95%CI, 1.26-7.29) was the only independent risk factor associated with grade 1-4 hepatotoxicity.

## Conclusion

Monitoring of hepatotoxicity should be considered in TB/HIV-1 co-infected patients who infected with HCV and receiving NVP.

